# Antibladder Cancer Effects of Excavatolide C by Inducing Oxidative Stress, Apoptosis, and DNA Damage In Vitro

**DOI:** 10.3390/ph15080917

**Published:** 2022-07-24

**Authors:** Che-Wei Yang, Tsu-Ming Chien, Chia-Hung Yen, Wen-Jeng Wu, Jyh-Horng Sheu, Hsueh-Wei Chang

**Affiliations:** 1 Graduate Institute of Natural Products, Kaohsiung Medical University, Kaohsiung 80708, Taiwan; u110531013@gap.kmu.edu.tw (C.-W.Y.); chyen@kmu.edu.tw (C.-H.Y.); 2 Graduate Institute of Clinical Medicine, College of Medicine, Kaohsiung Medical University, Kaohsiung 80756, Taiwan; u108801005@kmu.edu.tw (T.-M.C.); wejewu@kmu.edu.tw (W.-J.W.); 3 Department of Urology, Kaohsiung Medical University Hospital, Kaohsiung 80756, Taiwan; 4 Department of Urology, Faculty of Medicine, College of Medicine, Kaohsiung Medical University, Kaohsiung 80756, Taiwan; 5 Department of Marine Biotechnology and Resources, National Sun Yat-sen University, Kaohsiung 80424, Taiwan; 6 Department of Medical Research, China Medical University Hospital, China Medical University, Taichung 40402, Taiwan; 7 Center for Cancer Research, Kaohsiung Medical University, Kaohsiung 80708, Taiwan; 8 Department of Medical Research, Kaohsiung Medical University Hospital, Kaohsiung Medical University, Kaohsiung 80708, Taiwan; 9 Department of Biomedical Science and Environmental Biology, College of Life Science, Kaohsiung Medical University, Kaohsiung 80708, Taiwan

**Keywords:** soft coral, bladder cancer, apoptosis, oxidative stress, DNA damage

## Abstract

Excavatolide C (EXCC) is a bioactive compound derived from the gorgonian octocoral *Briareum excavatum*, and its anticancer effects are rarely addressed, particularly for bladder cancer. This investigation aimed to explore the potential impacts of EXCC on inhibiting the proliferation of three kinds of bladder cancer cells (5637, BFTC905, and T24). EXCC inhibits bladder cancer cell proliferation based on 48 h ATP assay. This antiproliferation function is validated to be oxidative stress dependent. Cellular and mitochondrial oxidative stresses were upregulated by EXCC, accompanied by depleting glutathione and mitochondrial membrane potential. These antiproliferation and oxidative stress events were suppressed by *N*-acetylcysteine (NAC), indicating that EXCC has an oxidative stress-regulating function for antiproliferation of bladder cancer cells. Oxidative stress-related responses such as apoptosis, caspase activation, and DNA damage were upregulated by EXCC and reverted by NAC. Taken together, the antiproliferation function of EXCC provides a potential treatment against bladder cancer cells via oxidative stress modulation.

## 1. Introduction

Bladder cancer was the eighth leading cause of cancer death according to the United States Cancer Statistics [1]. Its estimated new cases and deaths for both genders were 83,730 and 17,200 for bladder cancer patients [1]. Bladder tumors are commonly treated by radical cystectomy coupled with cisplatin-based chemotherapy [2,3]. However, cisplatin occasionally generates adverse effects [4,5]. Therefore, it is critical to identify more antiproliferation agents for bladder cancer treatment.

Marine corals containing different natural products [6,7] were reported for anticancer treatment [8,9,10,11]. The gorgonian soft coral *Briareum excavatum* (*B. excavatum*) is commonly screened for natural products. Several bioactive compounds were reported from *B. excavatum* [12,13,14,15,16,17,18], and some studies provide their chemical characterizations. In some studies, the antiproliferation effects against several cancer cells were investigated. However, they generally offered cytotoxicity with IC_50_ values and lacked investigations of detailed anticancer mechanisms.

The α,β-epoxy-γ-lactone motif commonly existed in several natural products with anticancer effects, such as lophotoxin [19] and bipinnatin Q [20], for antileukemia function. Several *B. excavatum*-derived excavatolide compounds, such as excavatolide B-E, contain this α,β-epoxy-γ-lactone motif. Excavatolide B is the most abundant *B. excavatum*-derived metabolite providing the generation of oxidative stress and antiproliferation in lung cancer cells [21]. However, the anticancer study for another excavatolide, namely excavatolide C (EXCC), was rarely investigated. EXCC was discovered in 1998 [12] and showed cytotoxicity (IC_50_ values) to lung and colon cancer cells. However, the anticancer mechanisms of EXCC remain unclear.

This investigation aims to evaluate the antiproliferation impacts of EXCC against bladder cancer cells. Anticancer mechanisms of oxidative stress were the particular objective, as well as associated changes, such as apoptosis and DNA damage to bladder cancer cells.

## 2. Results

### 2.1. Proliferation Change by EXCC

**Figure 1:**
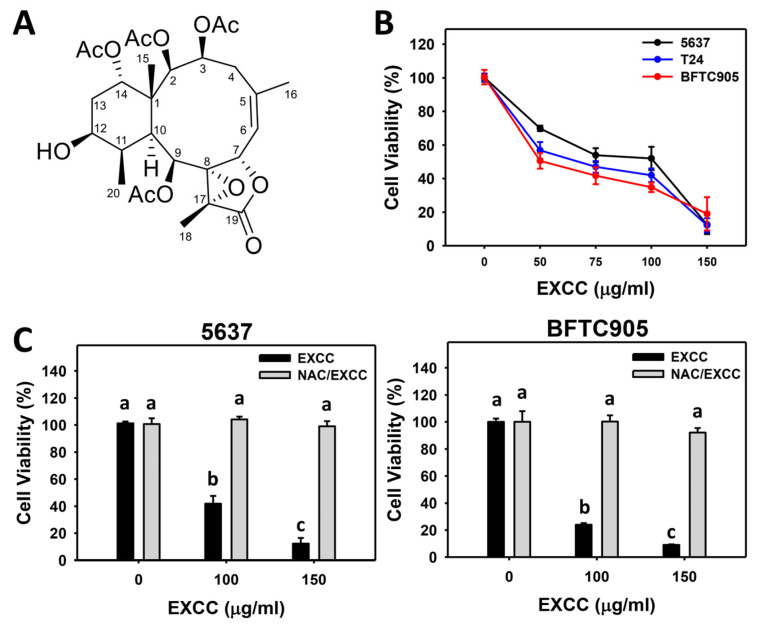
Proliferation change by EXCC. (**A**) Structure. (**B**) ATP assay of EXCC. Three bladder cancer cell lines (5637, BFTC905, and T24) were tested. Cells were treated with control (0.1% DMSO) and EXCC for 48 h and subjected to an ATP assay to determine cell viability. (**C**) ATP assay of NAC/EXCC. *N*-acetylcysteine (NAC)/EXCC is the NAC pretreatment (10 mM, 1 h) coupled with EXCC posttreatment (100 and 150 μg/mL, 48 h). Statistical lower-case letters were given to each treatment. Non-overlapping letters between different treatments indicate significant results (*p* < 0.05). Data, means ± SD (*n* = 3). For example (5637 cells in Figure 1C), the EXCC (black color) 0, 100, and 150 indicating “a, b, and c” show significant results between each other because these letters were non-overlapping. In contrast, EXCC 0 (black color) and NAC/EXCC (gray color) 0, 100, and 150 indicating “a” show nonsignificant results because these letters overlap.

The modulating impacts of proliferation of EXCC (Figure 1A) were tested using three kinds of bladder cancer cell lines (5637, BFTC905, and T24). EXCC at 48 h treatment dose-responsively reduced the cell viability of these bladder cancer cells (Figure 1B). To assess the effects of oxidative stress, an inhibitor of oxidative stress such as *N*-acetylcysteine (NAC) was pretreated before EXCC treatment, i.e., NAC/EXCC. Subsequently, antiproliferation effects exerted by EXCC were suppressed by NAC (NAC/EXCC), and their cell viabilities were recovered to similar levels as in the control (Figure 1C).

### 2.2. Cell Cycle Change by EXCC

**Figure 2:**
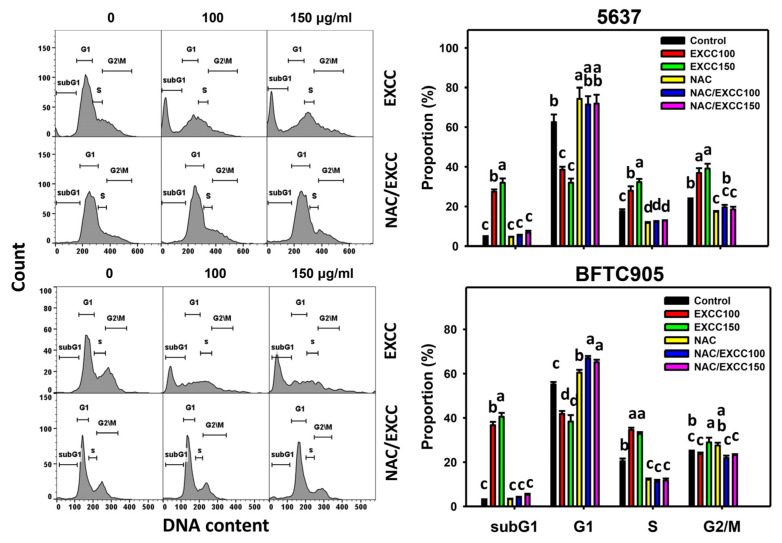
Cell cycle change by EXCC and NAC/EXCC. Two bladder cancer cell lines (5637 and BFTC905) were tested. Cells were treated with control (0.1% DMSO) and EXCC (100 and 150 μg/mL) for 48 h. NAC/EXCC is the NAC pretreatment (10 mM, 1 h) coupled with EXCC posttreatment (100 and 150 μg/mL, 48 h). Statistically different lower-case letters were given to each treatment. Non-overlapping letters between different treatments indicate significant results (*p* < 0.05). Data, means ± SD (*n* = 3). For the example of subG1 (5637 cells), the EXCC 0 (control), 100, and 150 indicating “c, b and a” show significant results between each other because these letters were non-overlapping. In contrast, NAC, NAC/EXCC 100, and NAC/EXCC 150 indicating “c” show nonsignificant results because these letters overlap.

EXCC at 48 h treatment upregulated the subG1 (%) of bladder cancer cells (5637 and BTFC905) (Figure 2). To evaluate the effect of oxidative stress in the cell cycle progression, NAC was pretreated before EXCC treatment. Subsequently, the subG1 increment exerted by EXCC was suppressed by NAC (NAC/EXCC), and their subG1 (%) was recovered similar to the control (Figure 2).

In addition to subG1 changes, EXCC affected other cell phases in bladder cancer cells. For 5637 bladder cancer cells, EXCC decreased the G1 phase and increased S and G2/M phases, which were reverted by NAC (Figure 2). For BTFC905 cells, EXCC decreased G1 and increased S phases, which NAC reverted.

### 2.3. Apoptosis (Annexin V) Change by EXCC

**Figure 3:**
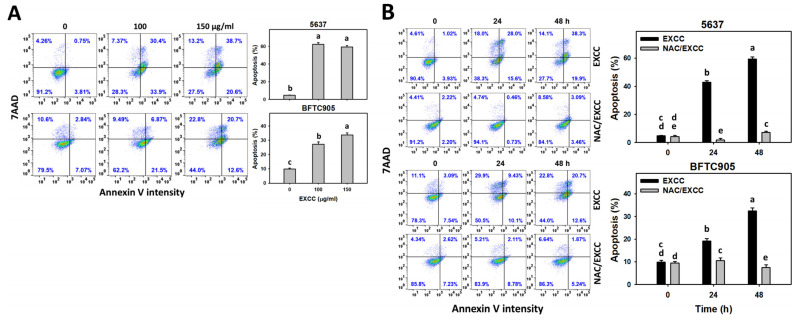
Apoptosis (annexin V) change by EXCC. (**A**) Annexin V assay of EXCC. Two bladder cancer cell lines (5637 and BFTC905) were tested. Cells were treated with control (0.1% DMSO) and EXCC (100 and 150 μg/mL) for 48 h. Apoptosis (%) was determined by calculation of annexin V (+)/7ADD (±) (%). (**B**) Annexin V assay of NAC/EXCC. NAC/EXCC was NAC pretreatment (10 mM, 1 h) coupled with EXCC posttreatment (100 and 150 μg/mL, 24 and 48 h). Statistical lower-case letters were given to each treatment. Non-overlapping letters between different treatments indicate significant results (*p* < 0.05). Data, means ± SD (*n* = 3). For example (5637 cells in Figure 3B), the EXCC (black color) 0, 24, and 48 h indicating “cd, b and a” show significant results between each other because these letters were not overlapping. In contrast, EXCC 0 (black color) and NAC/EXCC (gray color) 0 h indicating “cd and de” show nonsignificant results because these letters were overlapping with “d”.

Annexin V detection is one of the apoptosis indicators [22]. EXCC at 48 h treatment upregulated the annexin V (+) (%) of bladder cancer cells (5637 and BTFC905) (Figure 3A). To assess the participation of oxidative stress in apoptosis, NAC was pretreated before EXCC treatment. Subsequently, the annexin V (+) increment exerted by EXCC was suppressed by NAC (NAC/EXCC), and their annexin V (+) (%) was recovered similar to the control (Figure 3B).

### 2.4. Caspase 3 (Cas 3) Activation Change by EXCC

**Figure 4:**
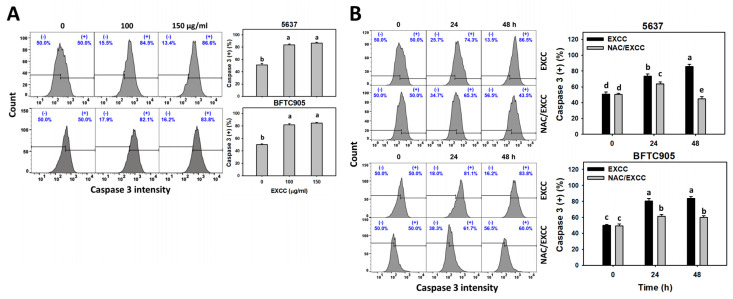
Cas 3 activation change by EXCC. (**A**) Cas 3 assays of EXCC. Two bladder cancer cell lines (5637 and BFTC905) were tested. Cells were treated with control (0.1% DMSO) and EXCC (100 and 150 μg/mL) for 48 h. (+) represents the Cas 3 (+) population. (**B**) Cas 3 assays of NAC/EXCC. NAC/EXCC is the NAC pretreatment (10 mM, 1 h) coupled with EXCC posttreatment (150 μg/mL, 24 and 48 h). Statistical lower-case letters were given to each treatment. Non-overlapping letters between different treatments indicate significant results (*p* < 0.05). Data, means ± SD (*n* = 3). For example (5637 cells in Figure 4B), the EXCC (black color) 0, 24, and 48 h indicating “d, b, and a” show significant results between each other because these letters were non-overlapping. In contrast, EXCC 0 (black color) and NAC/EXCC (gray color) 0 h indicating “d” show nonsignificant results because these letters overlap.

Cas 3 activation is one of the apoptosis indicators [23]. EXCC at 48 h treatment upregulated the Cas 3 (+) (%) of bladder cancer cells (5637 and BTFC905) (Figure 4A). To assess the participation of oxidative stress in apoptosis, NAC was pretreated before EXCC treatment. Subsequently, the Cas 3 (+) increment exerted by EXCC was suppressed by NAC (NAC/EXCC), and their Cas 3 (+) (%) was recovered like control (Figure 4B).

### 2.5. Caspase 8/9 (Cas 8/9) Changes by EXCC

**Figure 5:**
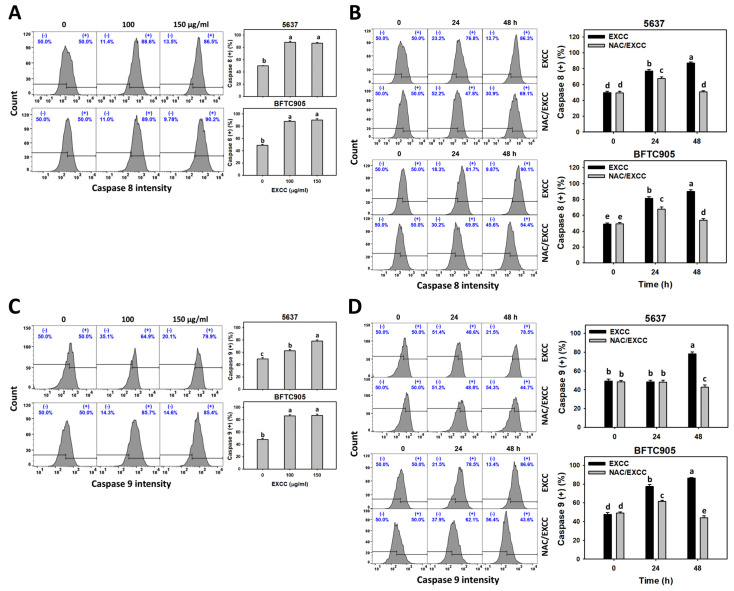
Cas 8 and Cas 9 changes by EXCC. (**A**,**C**) Cas 8 and Cas 9 assays of EXCC. Two bladder cancer cell lines (5637 and BFTC905) were tested. Cells were treated with control (0.1% DMSO) and EXCC (100 and 150 μg/mL) for 48 h. (+) represents the Cas 8 or Cas 9 (+) population. (**B**,**D**) Cas 8 and Cas 9 assays of NAC/EXCC. NAC/EXCC is the NAC pretreatment (10 mM, 1 h) coupled with EXCC posttreatment (150 μg/mL, 24 and 48 h). Statistical lower-case letters were given to each treatment. Non-overlapping letters between different treatments indicate significant results (*p* < 0.05). Data, means ± SD (*n* = 3). For example (5637 cells in Figure 5B), the EXCC (black color) 0, 24, and 48 h indicating “d, b and a” show significant results between each other because these letters were non-overlapping. In contrast, EXCC 0 (black color) and NAC/EXCC (gray color) 0 h indicating “d” show nonsignificant results because these letters overlap.

Cas 8/9 activations are extrinsic and intrinsic apoptosis indicators [23]. EXCC at 48 h treatment upregulated the Cas 8/9 (+) (%) of bladder cancer cells (5637 and BTFC905) (Figure 5A,C). To evaluate the participation of oxidative stress in apoptosis, NAC was pretreated before EXCC treatment. Subsequently, the Cas 8/9 (+) (%) increment exerted by EXCC was suppressed by NAC (NAC/EXCC), and their Cas 8/9 (+) (%) was recovered like control (Figure 5B,D).

### 2.6. Oxidative Stress Change by EXCC

The depletion effects of oxidative stress were tested with NAC (Figure 1, Figure 2, Figure 3, Figure 4 and Figure 5). However, the oxidative stress changes were not examined for EXCC treatment in bladder cancer cells. Using the specific probes for reactive oxygen species (ROS) and mitochondrial superoxide (MitoSOX) [24], different kinds of oxidative stresses were monitored.

**Figure 6:**
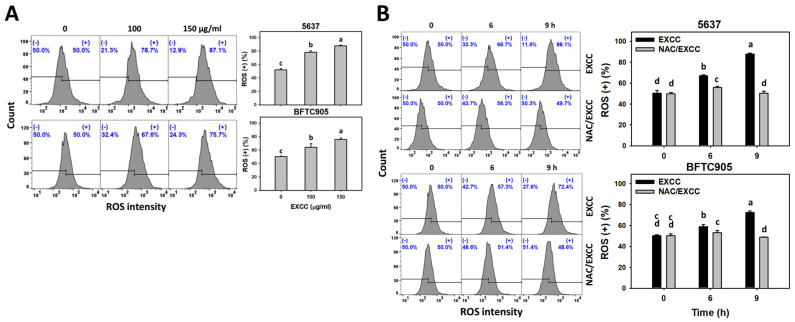
ROS change by EXCC. (**A**) ROS assay of EXCC. Two bladder cancer cell lines (5637 and BFTC905) were tested. Cells were treated with control (0.1% DMSO) and EXCC (100 and 150 μg/mL) for 9 h. (+) represents ROS (+) population. (**B**) ROS assay of NAC/EXCC. NAC/EXCC is the NAC pretreatment (10 mM, 1 h) coupled with EXCC posttreatment (150 μg/mL, 6 and 9 h). Statistical lower-case letters were given to each treatment. Non-overlapping letters between different treatments indicate significant results (*p* < 0.05). Data, means ± SD (*n* = 3). For example (5637 cells in Figure 6B), the EXCC (black color) 0, 6, and 9 h indicating “d, b, and a” show significant results between each other because these letters were non-overlapping. In contrast, EXCC 0 (black color) and NAC/EXCC (gray color) 0 h indicating “d” show nonsignificant results because these letters overlap.

**Figure 7:**
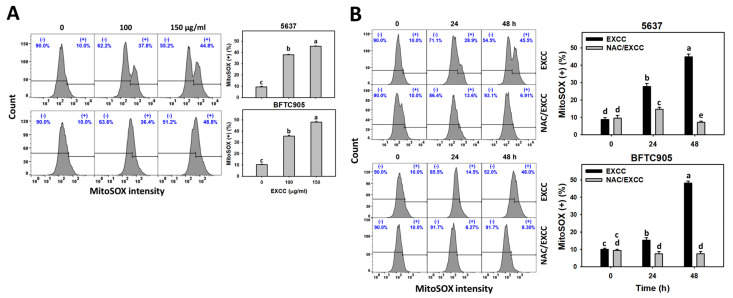
MitoSOX change by EXCC. (**A**) MitoSOX assay of EXCC. Two bladder cancer cell lines (5637 and BFTC905) were tested. Cells were treated with control (0.1% DMSO) and EXCC (100 and 150 μg/mL) for 48 h. (+) represents MitoSOX (+) population. (**B**) MitoSOX assay of NAC/EXCC. NAC/EXCC is the NAC pretreatment (10 mM, 1 h) coupled with EXCC posttreatment (150 μg/mL, 24 and 48 h). Statistical lower-case letters were given to each treatment. Non-overlapping letters between different treatments indicate significant results (*p* < 0.05). Data, means ± SD (*n* = 3). For example (5637 cells in Figure 7B), the EXCC (black color) 0, 24, and 48 h indicating “d, b, and a” show significant results between each other because these letters were non-overlapping. In contrast, EXCC 0 (black color) and NAC/EXCC (gray color) 0 h indicating “d” show nonsignificant results because these letters overlap.

EXCC at 9 and 48 h treatments upregulated the ROS and MitoSOX (+) (%) of bladder cancer cells (5637 and BTFC905) (Figure 6A and Figure 7A). To evaluate the participation of oxidative stress, NAC was pretreated before EXCC treatment. Subsequently, the ROS and MitoSOX (+) (%) increment exerted by EXCC was suppressed by NAC (NAC/EXCC), and their ROS and MitoSOX (+) (%) was recovered similar to the control (Figure 6B and Figure 7B).

**Figure 8:**
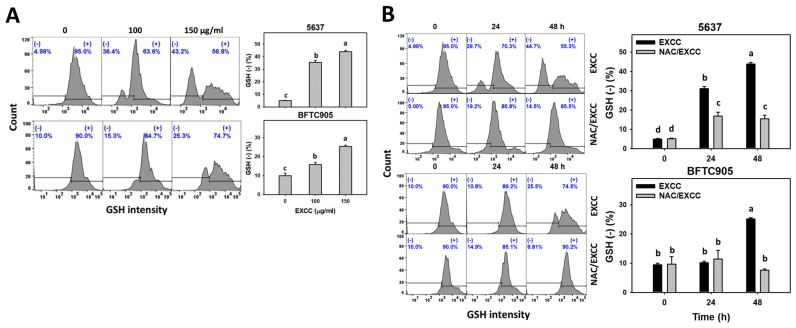
GSH change by EXCC. (**A**) GSH assay of EXCC. Two bladder cancer cell lines (5637 and BFTC905) were tested. Cells were treated with control (0.1% DMSO) and EXCC (100 and 150 μg/mL) for 48 h. (−) represents GSH (−) population. (**B**) GSH assay of NAC/EXCC. NAC/EXCC is the NAC pretreatment (10 mM, 1 h) coupled with EXCC posttreatment (150 μg/mL, 24 and 48 h). Statistical lower-case letters were given to each treatment. Non-overlapping letters between different treatments indicate significant results (*p* < 0.05). Data, means ± SD (*n* = 3). For example (5637 cells in Figure 8B), the EXCC (black color) 0, 24, and 48 h indicating “d, b, and a” show significant results between each other because these letters were non-overlapping. In contrast, EXCC 0 (black color) and NAC/EXCC (gray color) 0 h indicating “d” show nonsignificant results because these letters overlap.

EXCC at 48 h treatment upregulated the glutathione (GSH) (−) (%) of bladder cancer cells (5637 and BTFC905) (Figure 8A). To evaluate the participation of oxidative stress, NAC was pretreated before EXCC treatment. Subsequently, the GSH (−) (%) increment exerted by EXCC was suppressed by NAC (NAC/EXCC), and their GSH (−) (%) was recovered similar to the control (Figure 8B).

### 2.7. Mitochondrial Membrane Potential (MMP) Change by EXCC

**Figure 9:**
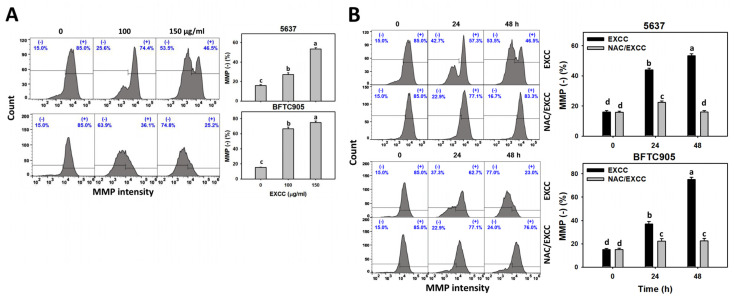
MMP change by EXCC. (**A**) MMP assay of EXCC. Two bladder cancer cell lines (5637 and BFTC905) were tested. Cells were treated with control (0.1% DMSO) and EXCC (100 and 150 μg/mL) for 48 h. (−) represents MMP (−) population. (**B**) MMP assay of NAC/EXCC. NAC/EXCC is the NAC pretreatment (10 mM, 1 h) coupled with EXCC posttreatment (150 μg/mL, 24 and 48 h). Statistical lower-case letters were given to each treatment. Non-overlapping letters between different treatments indicate significant results (*p* < 0.05). Data, means ± SD (*n* = 3). For example (5637 cells in Figure 9B), the EXCC (black color) 0, 24, and 48 h indicating “d, b, and a” show significant results between each other because these letters were non-overlapping. In contrast, EXCC 0 (black color) and NAC/EXCC (gray color) 0 h indicating “d” show nonsignificant results because these letters overlap.

MMP is another indicator for evaluating oxidative stress [25,26]. EXCC at 48 h treatment upregulated the MMP (−) (%) of bladder cancer cells (5637 and BTFC905) (Figure 9A). To evaluate the participation of oxidative stress, NAC was applied before EXCC treatment. Subsequently, the MMP (−) (%) increment exerted by EXCC was suppressed by NAC (NAC/EXCC), and their MMP (−) (%) was recovered similar to the control (Figure 9B).

### 2.8. DNA Damage Change by EXCC

**Figure 10:**
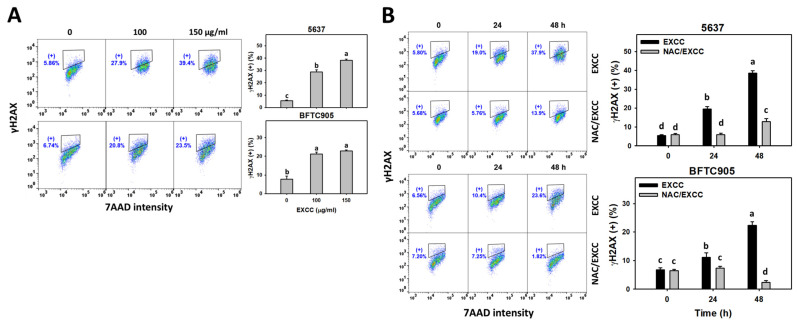
γH2AX change by EXCC. (**A**) γH2AX assay of EXCC. Two bladder cancer cell lines (5637 and BFTC905) were tested. Cells were treated with solvent control (0.1% DMSO) and EXCC (100 and 150 μg/mL) for 48 h. (+) represents the γH2AX (+) population. (**B**) γH2AX assay of NAC/EXCC. NAC/EXCC is the NAC pretreatment (10 mM, 1 h) coupled with EXCC posttreatment (150 μg/mL, 24 and 48 h). Statistical lower-case letters were given to each treatment. Non-overlapping letters between different treatments indicate significant results (*p* < 0.05). Data, means ± SD (*n* = 3). For example (5637 cells in Figure 10B), the EXCC (black color) 0, 24, and 48 h indicating “d, b, and a” show significant results between each other because these letters were non-overlapping. In contrast, EXCC 0 (black color) and NAC/EXCC (gray color) 0 h indicating “d” show nonsignificant results because these letters overlap.

**Figure 11:**
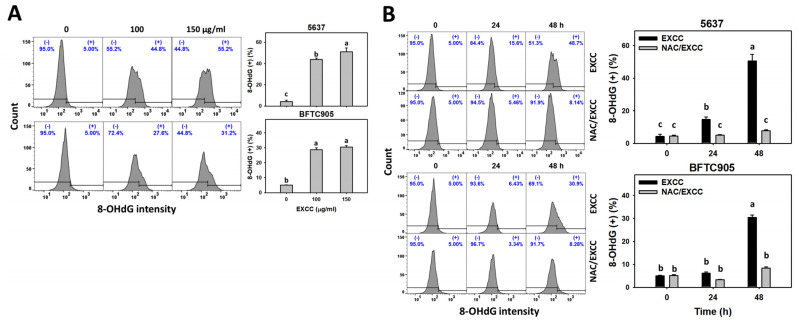
8-OHdG change by EXCC. (**A**) 8-OHdG assay of EXCC. Two bladder cancer cell lines (5637 and BFTC905) were tested. Cells were treated with control (0.1% DMSO) and EXCC (100 and 150 μg/mL) for 48 h. (+) represents the 8-OHdG (+) population. (**B**) 8-OHdG assay of NAC/EXCC. NAC/EXCC is the NAC pretreatment (10 mM, 1 h) coupled with EXCC posttreatment (150 μg/mL, 24 and 48 h). Statistical lower-case letters were given to each treatment. Non-overlapping letters between different treatments indicate significant results (*p* < 0.05). Data, means ± SD (*n* = 3). For example (5637 cells in Figure 11B), the EXCC (black color) 0, 24, and 48 h indicating “c, b, and a” show significant results between each other because these letters were non-overlapping. In contrast, EXCC 0 (black color) and NAC/EXCC (gray color) 0 h indicating “c” show nonsignificant results because these letters overlap.

EXCC at 48 h treatment upregulated the γH2AX and 8-hydroxy-2′-deoxyguanosine (8-OHdG) (+) (%) of bladder cancer cells (5637 and BTFC905) (Figure 10A and Figure 11A). To evaluate the participation of oxidative stress, NAC was pretreated before EXCC treatment. Subsequently, the γH2AX and 8-OHdG (+) (%) increment exerted by EXCC was suppressed by NAC (NAC/EXCC), and their γH2AX and 8-OHdG (+) (%) was recovered similar to the control (Figure 10B and Figure 11B).

## 3. Discussion

*B. excavatum*-derived EXCC showed cytotoxicity to lung and colon cancer cells. However, the anticancer mechanisms of EXCC remain unclear. The present study validates this hypothesis and confirms that EXCC induces apoptosis and DNA damage to bladder cancer cells.

### 3.1. Comparison of Antiproliferation of EXCC in Different Cancer Cell Lines

The IC_50_ value of EXCC for lung (A549) and colon (HT-29) cancer cells were 1.9 μg/mL, according to 72 h MTT assays [12]. In the present study, the IC_50_ values of EXCC for bladder (BFTC905, T24, and 5637) cancer cells were 51, 62, and 100 μg/mL according to a 48 h ATP assay (Figure 1). Accordingly, EXCC showed different drug sensitivities to various cancer cells.

For comparison, cisplatin, a common clinical drug for bladder cancer, showed IC_50_ values of 1.33 and 4.98 μg/mL for T24 and J82 cells at 48 h CCK-8 assay, respectively [27]. Cisplatin also showed IC_50_ values of 4.79, 8.61, 38.54, and 27.85 μg/mL for 5637, J82, HT119, and 253J cells at 96 h trypan blue assay, respectively [28]. Notably, cisplatin showed severe side effects in clinical use [29]. It warrants a detailed assessment of the cytotoxicity of a non-cancer cell line to evaluate the selectivity of EXCC in the future.

### 3.2. Role of Oxidative Stress in Antiproliferation of EXCC 

Oxidative stress-generating drugs commonly inhibit cancer cell proliferation [30,31,32,33]. For example, cryptocaryone triggers ROS and apoptosis to inhibit the proliferation of ovarian cancer cells [34]. A marine natural product such as fucoidan can upregulate oxidative stress and consequently induce apoptosis and cause antiproliferation of oral cancer cells [33]. Excavatolide B induces ROS and RNS production, leading to antiproliferation in lung cancer cells [21]. After examination, EXCC caused antiproliferation, cellular and mitochondrial oxidative stress such as ROS and MitoSOX, and MMP depletion in bladder cancer cells (Figure 6, Figure 7 and Figure 9). These antiproliferation (Figure 1C) and oxidative stresses were suppressed by NAC, suggesting that EXCC exerts an oxidative stress-mediated antiproliferation to bladder cancer cells.

Moreover, redox homeostasis is balanced by oxidants and antioxidants [35,36]. GSH is one of the cellular antioxidants that downregulates oxidative stress [37,38]. Accordingly, GSH depletion leads to increased oxidative stress [31]. For example, fucoidan shows oxidative stress in oral cancer cells accompanied by GSH depletion. EXCC demonstrates a similar result in bladder cancer cells (Figure 8). Accordingly, EXCC downregulates GSH to trigger oxidative stress in bladder cancer cells.

### 3.3. Apoptosis and DNA Damage Effects of EXCC Involving Oxidative Stress

Oxidative stress may evoke several cellular responses, such as apoptosis [39] and DNA damage [31]. For example, cryptocaryone-induced ROS promotes apoptosis and DNA damage in ovarian cancer cells [34]. Similarly, EXCC triggers apoptosis as evidenced by the cell cycle, annexin V, and caspase signaling (Figure 2, Figure 3 and Figure 4). The present study showed that EXCC increases subG1 events of bladder cancer cells (Figure 2), which is the apoptosis-like changes. This is further validated by annexin V detection (Figure 3). Consistently, EXCC also activates apoptosis executor caspase 3 (Figure 4). Moreover, EXCC triggered both extrinsic and intrinsic caspase activations such as caspases 8 and 9 in bladder cancer cells (Figure 5). These apoptosis changes and signaling activations were suppressed by NAC, suggesting that EXCC triggers oxidative stress-mediated apoptosis in bladder cancer cells.

In addition, EXCC promotes DNA damage as evidenced by upregulating γH2AX and 8-OHdG expressions for DNA double-strand breaks and oxidative DNA damage in bladder cancer cells (Figure 10 and Figure 11), which were suppressed by NAC, suggesting that EXCC exerts oxidative stress-mediated DNA damage to bladder cancer cells.

## 4. Materials and Methods

### 4.1. Extraction and Separation of EXCC

The gorgonian *B. excavatum* was harvested, freeze-dried, and minced as detailed in our previous study [12]. Then, the materials were extracted repeatedly by ethyl acetate (EtOAc). The combined organic extract was processed for evaporation and a dark green residue was generated. This was dissolved in EtOAc and then stored at 0 °C to provide a solid, representing a mixture of long-chained esters. After discarding this solid, the remaining mixture was purified by Si gel column chromatography, using hexane and hexane-EtOAc mixtures of increasing polarity. EXCC was obtained from a fraction eluted with hexane-EtOAc (3:1–2:1), which was further confirmed to be pure by melting point analyses (134–135 °C) and data from NMR spectroscopy (Supplementary Figures S1–S7).

### 4.2. Oxidative Stress Inhibitor

Pretreatment with NAC (10 mM, 1 h) (Sigma-Aldrich, St. Louis, MO, USA) [40,41,42] was applied to inhibit oxidative stress to examine its role in EXCC-induced changes.

### 4.3. Cell Culture and Viability

Three human bladder cancer cell lines were purchased from ATCC (Manassas, VA, USA) and Bioresource Collection and Research Center (BCRC) (Hsinchu, Taiwan), i.e., ATCC: 5637, T24, and BCRC: BFTC905. The culture medium was obtained from Roswell Park Memorial Institute (RPMI) medium (Gibco, Grand Island, NY, USA), containing 10% fetal bovine serum (FBS), 100 U/mL penicillin, and 100 μg/mL streptomycin. Cell viability was estimated by an ATP kit (PerkinElmer Life Sciences, Boston, MA, USA) following the user manual’s instructions.

### 4.4. Cell Cycle Assays

Following fixation with 75% ethanol, cells were stained by 7-aminoactinmycin D (Biotium, Inc., Hayward, CA, USA) (7AAD; 1 μg/mL, 30 min) [43] and analyzed by Guava easyCyte flow cytometry (Luminex, Austin, TX, USA). The cell cycle phases were determined by their different DNA contents.

### 4.5. Apoptosis (Annexin V/7AAD)

Annexin V/7AAD reagents (Strong Biotech Inc., Taipei, Taiwan), containing Annexin V-FITC and 7AAD at the final condition (1:1000 and 1 μg/mL), were incubated with cells for 30 min to detect apoptosis [33,44] according to the user’s manual. Finally, cells were analyzed by flow cytometry.

### 4.6. Apoptosis (Cas 3, 8, 9)

In addition to the apoptosis executor Cas 3, the extrinsic and intrinsic apoptosis caspases (Cas 8 and Cas 9) were assessed by OncoImmunin kits (Gaithersburg, MD, USA) [45,46]. PhiPhiLux-G1D2, CaspaLux8-L1D2, and CaspaLux9-M1D2, the specific substrates for Cas 3, Cas 8, and Cas 9, were mixed with cell suspensions (1:1000) at 37 °C for 1 h. The activated forms of Cas 3, Cas 8, and Cas 9 can cleave these substrates to generate green fluorescence, analyzed by flow cytometry.

### 4.7. ROS, MitoSOX, and GSH

ROS, MitoSOX, and GSH detecting reagents such as 2′,7′-dichlorodihydrofluorescein diacetate (DCFH-DA) [33,47], MitoSOX™ Red [48], and 5-chloromethylfluorescein diacetate (CMF-DA) [49] were utilized to monitor oxidative stress. They were purchased commercially, i.e., Molecular Probes, Invitrogen, Eugene, OR, USA; Sigma-Aldrich, St. Louis, MO, USA; and Thermo Fisher Scientific, Carlsbad, CA, USA, respectively. The final conditions for DCFH-DA, MitoSOX™ Red, and CMF-DA were 2, 5, and 5 μM for 30 min, respectively. Finally, the cells were analyzed by flow cytometry.

### 4.8. MMP

MMP detecting reagents, such as DiOC_2_ (3), were utilized to monitor oxidative stress [33]. It was purchased from Invitrogen (San Diego, CA, USA). The final condition for DiOC_2_ (3) was 50 nM for 20 min. Finally, the cells were analyzed by flow cytometry.

### 4.9. γH2AX

After fixation, the γH2AX-detecting antibody was utilized to monitor DNA double-strand breaks [33]. It was purchased from Santa Cruz Biotechnology (Santa Cruz, CA, USA). The final condition for p-histone H2A.X primary antibody was 500× dilution. The secondary antibody, Alexa Fluor^®^488 (Cell Signaling Technology, Beverly, MA, USA), was then added in the presence of 7AAD for 30 min incubation. Finally, the cells were analyzed by flow cytometry.

### 4.10. 8-OHdG

After fixation, 8-OHdG-detecting antibody-FITC (Santa Cruz Biotechnology) was utilized to monitor oxidative DNA damage [33]. Finally, the cells were analyzed by flow cytometry.

### 4.11. Statistical Analysis

JMP12 software (SAS Institute, Cary, NC, USA) was used to perform ANOVA with Tukey HSD test. JMP assigns lower-case letters to each treatment. Treatments without overlapping letters were judged to have significant results for multi-comparisons *p* < 0.05.

## 5. Conclusions

The anticancer effects of a *Briareum excavatum*-derived bioactive compound EXCC are rarely addressed, particularly for bladder cancer. By the examination of bladder cancer cells, we validated that EXCC is a promising antiproliferation agent for bladder cancer cells in an oxidative stress-dependent manner. Several examinations such as ROS and MitoSOX supported that EXCC exerts the mechanism of oxidative stress in bladder cancer cells. EXCC also induces another oxidative stress response, such as MMP depletion. Moreover, the cellular antioxidant GSH was downregulated by EXCC, validating the modulating ability of oxidative stress by EXCC. These oxidative stress-related changes such as ROS, MitoSOX, MMP, and GSH were reverted by NAC pretreatment, indicating that EXCC is an oxidative stress-generating anticancer agent to bladder cancer cells. EXCC-promoted oxidative stress is associated with apoptosis, extrinsic and intrinsic caspase signaling, and γH2AX and 8-OHdG DNA damage reverted by NAC pretreatment. In conclusion, EXCC is firstly validated to exhibit antiproliferation against bladder cancer cells, accompanied by an oxidative stress-associated mechanism.

## Data Availability

Data is contained within the article and supplementary materials.

## Supplementary Materials

The following are available online at https://www.mdpi.com/article/10.3390/ph15080917/s1, Figure S1: ^1^H NMR of EXCC measured at 28 °C (400 MHz, Me_2_CO-d*_6_*), Figure S2: ^1^H NMR of EXCC measured at 0 °C (400 MHz, Me_2_CO-d*_6_*), Figure S3: ^1^H NMR of EXCC measured at −20 °C (400 MHz, Me_2_CO-d*_6_*), Figure S4: ^1^H NMR of EXCC measured at −40 °C (400 MHz, Me_2_CO-d*_6_*), Figure S5: ^1^H NMR of EXCC measured at −60 °C (400 MHz, Me_2_CO-d*_6_*), Figure S6: ^1^H NMR of EXCC measured at −80 °C (400 MHz, Me_2_CO-d*_6_*), Figure S7: ^13^C NMR of EXCC measured at −60 °C (100 MHz, Me_2_CO-d*_6_*). References [12,50] are cited in the supplementary materials.
