# Supplementary material for: Antibladder Cancer Effects of Excavatolide C by Inducing Oxidative Stress, Apoptosis, and DNA Damage In Vitro
Source: Pharmaceuticals (Basel). 2022 Jul 24;15(8):917. doi: 10.3390/ph15080917 (PMC9329968; doi:10.3390/ph15080917)
Supplement: Supplementary file 1 [file pharmaceuticals-15-00917-s001.zip › pharmaceuticals-1801269-Supplementary.pdf]

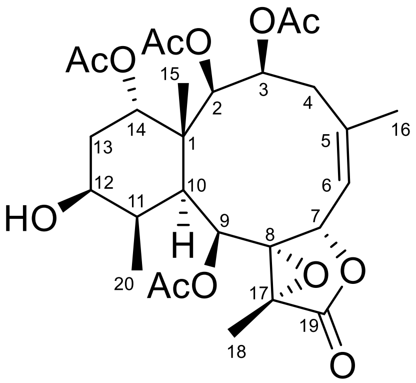

The purity of EXCC was proved by ^{1}H NMR measurements on various temperatures from 28 °C to -80 °C in Me_2CO-d_6. The measurement at 28 °C (Supplementary Figure S1, room temperature) shown broad and weak signals of cyclohexane ring (1.9–1.5 ppm), 10-membered carbocyclic ring (5.6–5.1 ppm), and acetoxyl groups (2.4–2.1 ppm); however, they can be regarded to be arisen from one compound. These observations suggested the existence of interconverting conformations of this compound in solution at room temp. Therefore, the ^{1}H and ^{13}C NMR measurements of EXCC were conducted below 0 °C to obtain well-resolved spectra with stable conformations (Supplementary Figures S2-S7). The lower temperature ^{1}H NMR measurement, especially at -60 and -80 °C (Supplementary Figures S5 and S6), displayed sharp signals and could be unambiguously assigned, together with minor signals from different conformers of the same compound due to a macro-ring (10-member ring) system. The rate for the conversion of various conformers could be reduced effectively by measuring at lower temperature, making the assignments of NMR signals of the stabilized conformers more likely [1, 2]. Therefore, the purity of EXCC could be concluded over 95%. This phenomenon had been found by the group of the corresponding author in 1998 [1].

References

1. Sheu, J.H.; Sung, P.J.; Cheng, M.C.; Liu, H.Y.; Fang, L.S.; Duh, C.Y.; Chiang, M.Y. Novel cytotoxic diterpenes, excavatolides A–E, isolated from the Formosan gorgonian *Briareum excavatum*. *J Nat Prod* **1998**, *61*, 602–608.
2. Sung, P.J.; Lin, M.R.; Su, Y.D.; Chiang, M.Y.; Hu, W.P.; Su, J.H.; Cheng, M.C.; Hwang, T.L.; Sheu, J.H. New briaranes from the octocorals *Briareum excavatum* (Briareidae) and *Junceella fragilis* (Ellisellidae). *Tetrahedron* **2008**, *64*, 2596–2604.

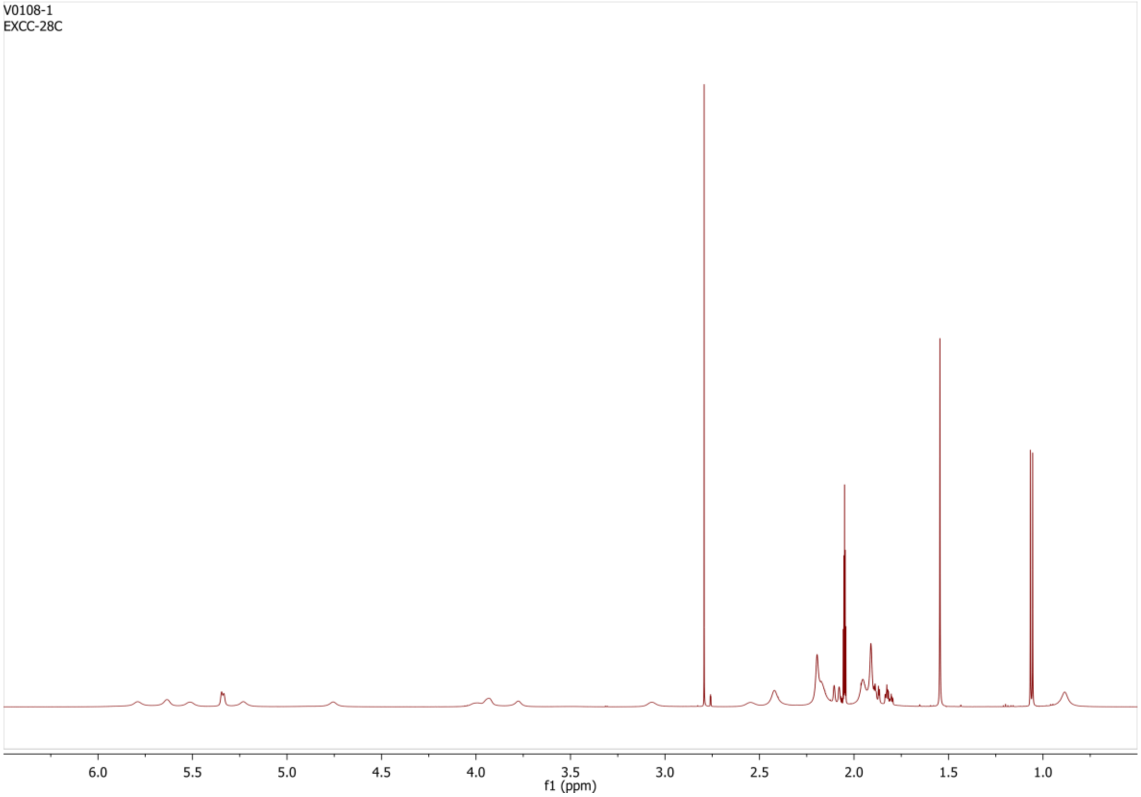

Supplementary Figure S1. ^{1}H NMR of EXCC measured at 28 °C (400 MHz, Me_2CO-d_6).

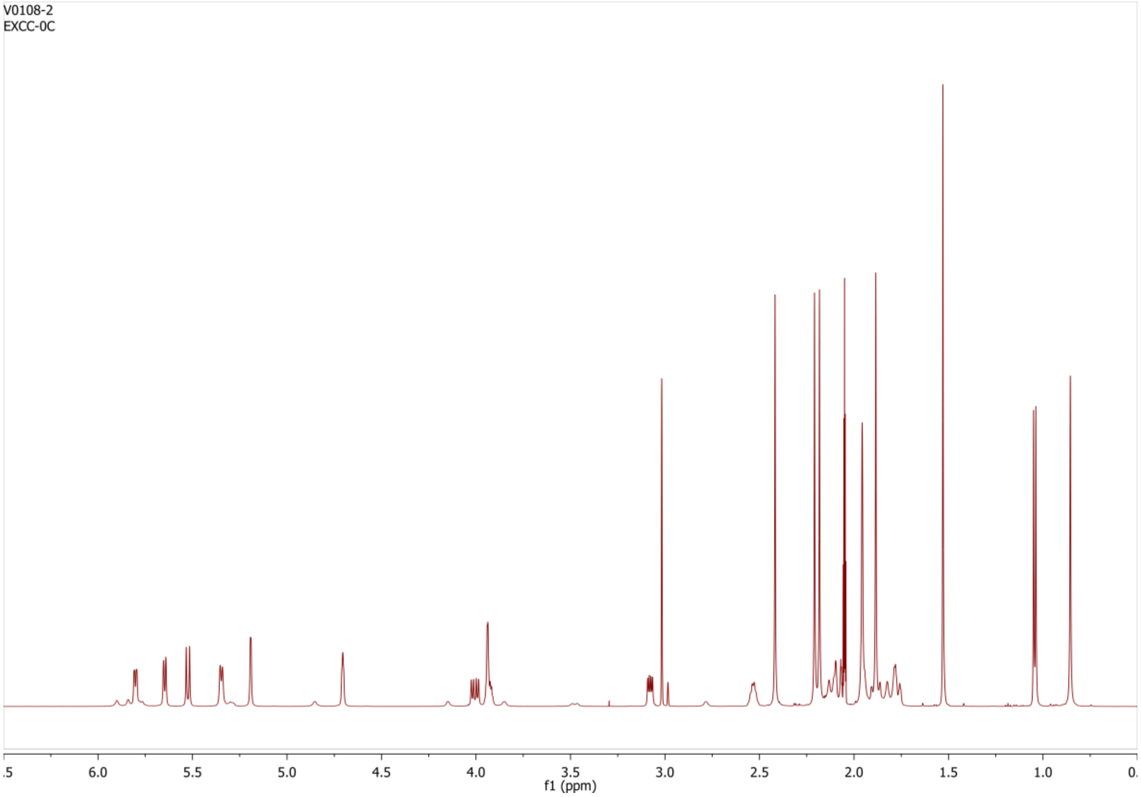

Supplementary Figure S2. ^{1}H NMR of EXCC measured at 0 °C (400 MHz, Me_2CO-d_6).

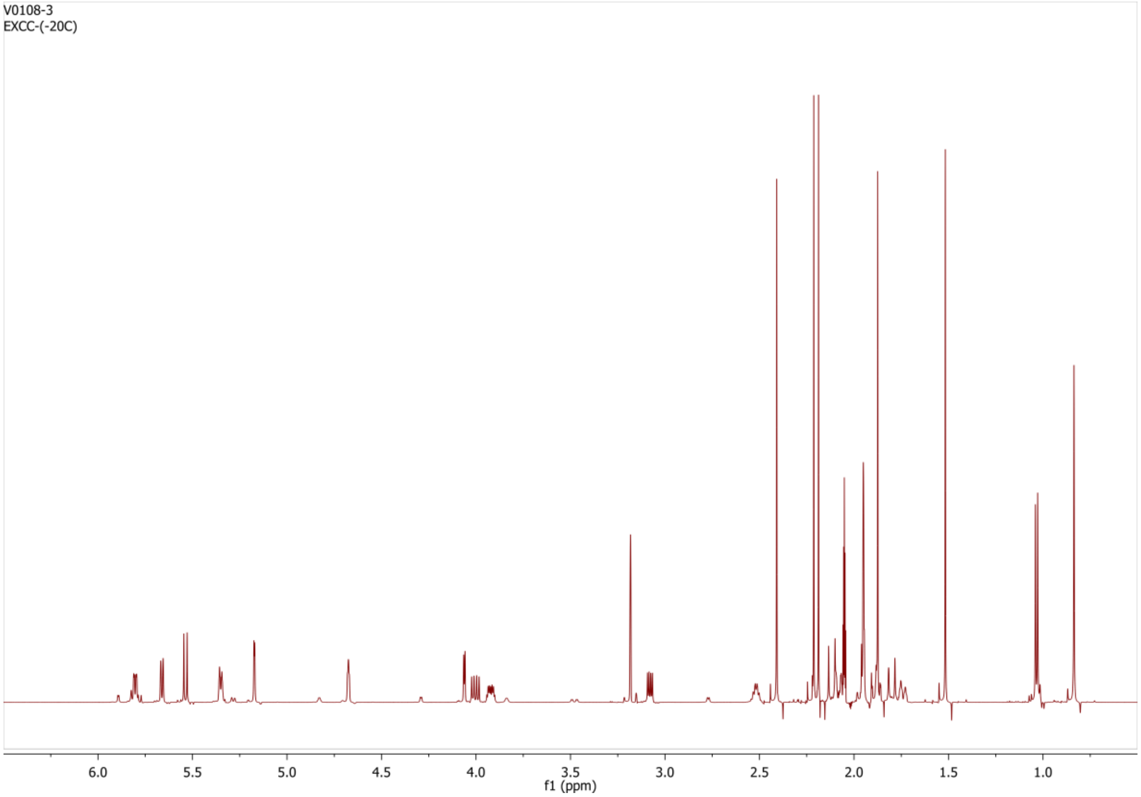

Supplementary Figure S3. ^{1}H NMR of EXCC measured at -20 °C (400 MHz, Me_2CO-d_6).

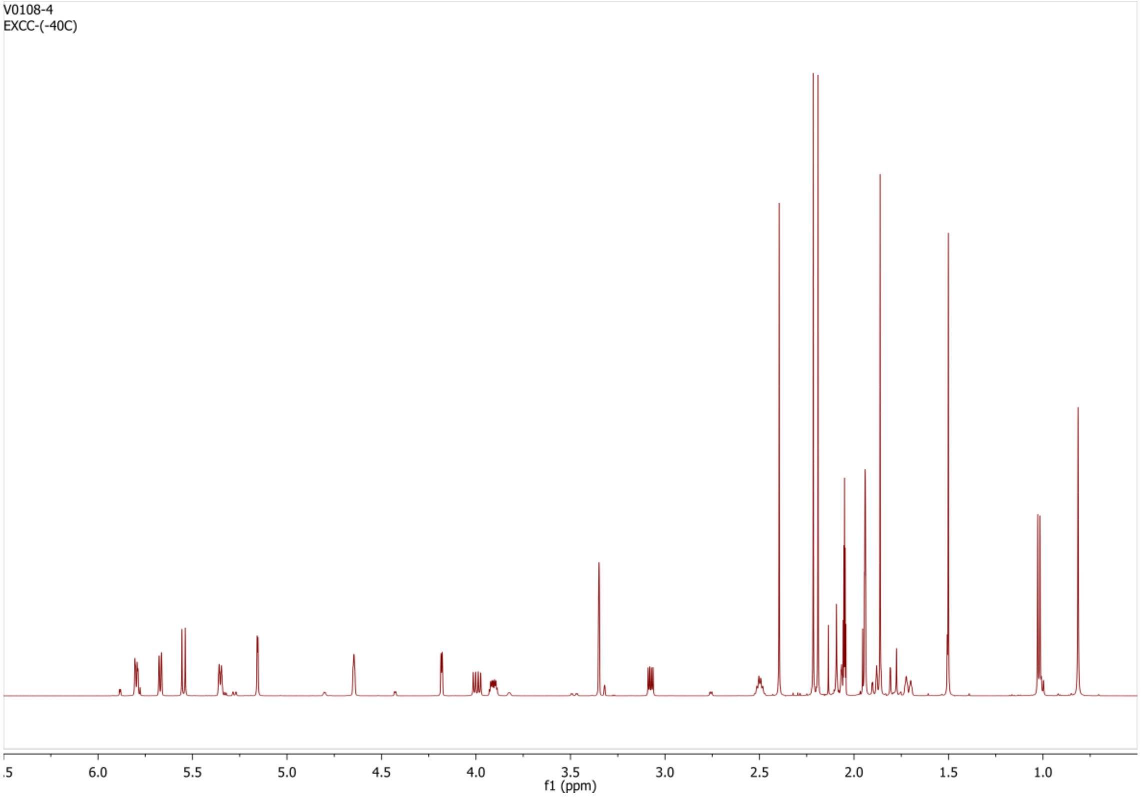

Supplementary Figure S4. ^{1}H NMR of EXCC measured at -40 °C (400 MHz, Me_2CO-d_6).

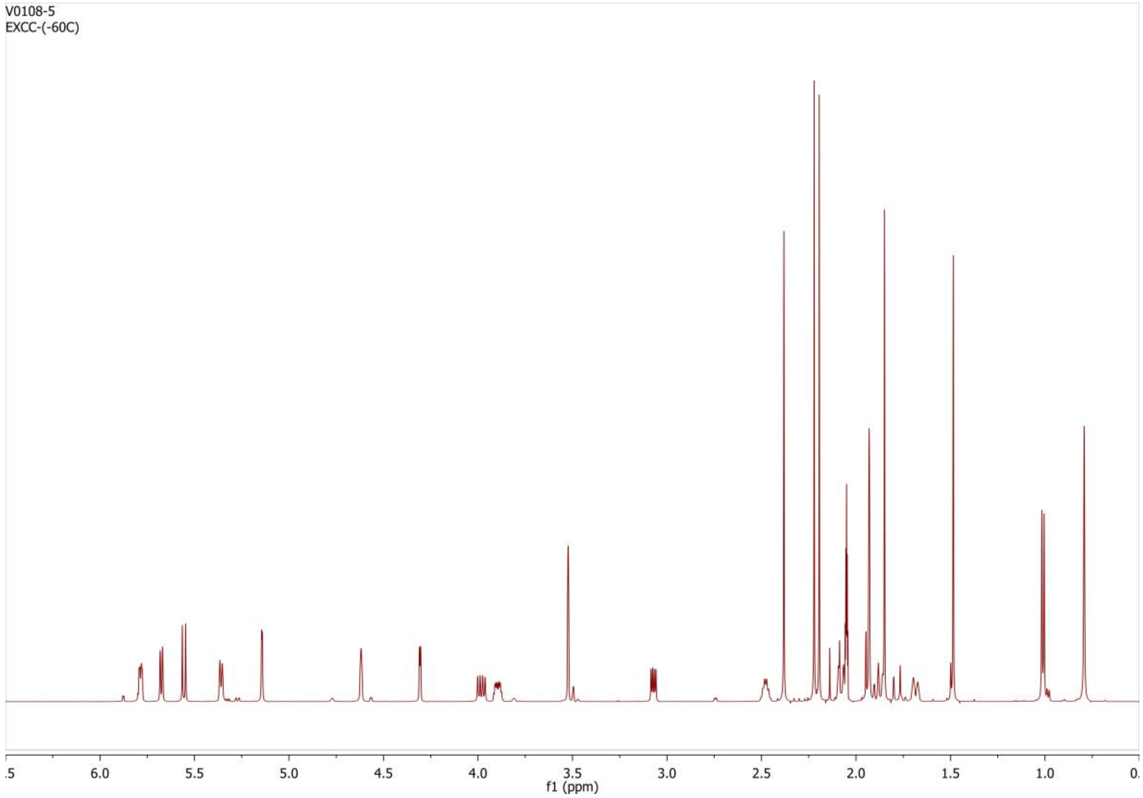

Supplementary Figure S5. ^{1}H NMR of EXCC measured at -60 °C (400 MHz, Me_2CO-d_6).

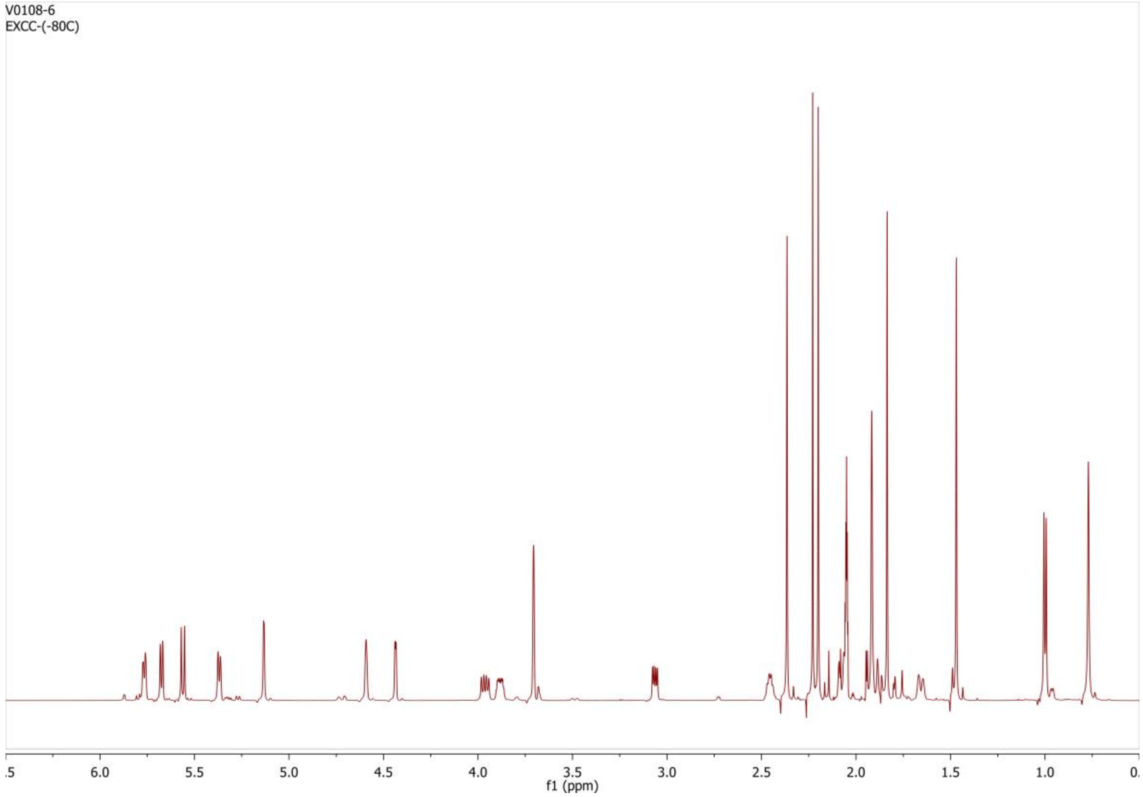

Supplementary Figure S6. ^{1}H NMR of EXCC measured at -80 °C (400 MHz, Me_2CO-d_6).

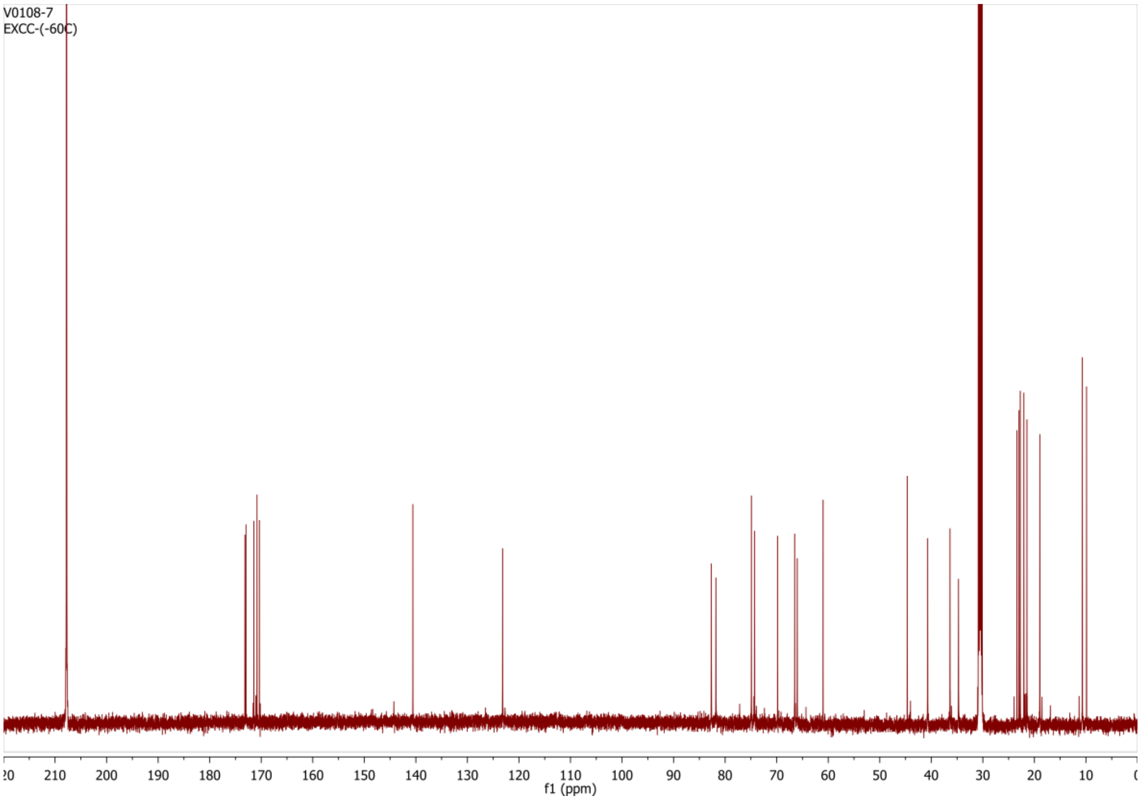

Supplementary Figure S7. ^{13}C NMR of EXCC measured at -60 °C (100 MHz, Me_2CO-d_6).
